# Single-Cell Transcriptome Sequencing Reveals Molecular Expression Differences and Marker Genes in Testes during the Sexual Maturation of Mongolian Horses

**DOI:** 10.3390/ani14091258

**Published:** 2024-04-23

**Authors:** Yuanyi Liu, Ming Du, Xinyu Li, Lei Zhang, Bilig Zhao, Na Wang, Manglai Dugarjaviin

**Affiliations:** 1 Key Laboratory of Equus Germplasm Innovation, Ministry of Agriculture and Rural Affairs, Hohhot 010018, China; 13470105913@163.com (Y.L.); duming@imau.edu.cn (M.D.); chinalxy94@163.com (X.L.); 13034784525@163.com (L.Z.); bilig9@163.com (B.Z.); 15804814519@163.com (N.W.); 2 Inner Mongolia Key Laboratory of Equine Science Research and Technology Innovation, Inner Mongolia Agricultural University, Hohhot 010018, China; 3 Equus Research Center, Inner Mongolia Agricultural University, Hohhot 010018, China

**Keywords:** Mongolian horse, sexually immature, sexually mature, testis, seminiferous tubules, single-cell transcriptome sequencing

## Abstract

**Simple Summary:**

The results of this research revealed significant differences in the tissue morphology and gene expression profiles of Mongolian horses based on age. Specifically, we identified 25 cell clusters and 10 cell types, including spermatogonial and somatic cells, from 17,000 testicular cells of Mongolian horses, constructing a single-cell atlas of the testes. Differential gene expression analysis highlighted distinct patterns in sexually immature and mature testes, with genes related to cellular infrastructure predominating in the former and those associated with spermatogenesis in the latter. Furthermore, we identified marker genes specific to each developmental stage, including *APOA1*, *AMH*, *TAC3*, *INHA*, *SPARC*, and *SOX9* for the sexually immature stage, and *PRM1*, *PRM2*, *LOC100051500*, *PRSS37*, *HMGB4*, and *H1-9* for the sexually mature stage.

**Abstract:**

This study aimed to investigate differences in testicular tissue morphology, gene expression, and marker genes between sexually immature (1-year-old) and sexually mature (10-year-old) Mongolian horses. The purposes of our research were to provide insights into the reproductive physiology of male Mongolian horses and to identify potential markers for sexual maturity. The methods we applied included the transcriptomic profiling of testicular cells using single-cell sequencing techniques. Our results revealed significant differences in tissue morphology and gene expression patterns between the two age groups. Specifically, 25 cell clusters and 10 cell types were identified, including spermatogonial and somatic cells. Differential gene expression analysis highlighted distinct patterns related to cellular infrastructure in sexually immature horses and spermatogenesis in sexually mature horses. Marker genes specific to each stage were also identified, including *APOA1*, *AMH*, *TAC3*, *INHA*, *SPARC*, and *SOX9* for the sexually immature stage, and *PRM1*, *PRM2*, *LOC100051500*, *PRSS37*, *HMGB4*, and *H1-9* for the sexually mature stage. These findings contribute to a deeper understanding of testicular development and spermatogenesis in Mongolian horses and have potential applications in equine reproductive biology and breeding programs. In conclusion, this study provides valuable insights into the molecular mechanisms underlying sexual maturity in Mongolian horses.

## 1. Introduction

Male Mongolian horse fertility is closely related to spermatogenesis. Spermatogenesis is a complex reproductive process with dynamics and continuity in which multiple spermatogenic epithelial and somatic cells are involved; it plays a crucial role in animal reproduction [1,2,3]. Disorders of spermatogenesis will lead to low ejaculate volume, azoospermia, oligozoospermia, necrospermia, sperm deformities, and low sperm motility in male animals, which will further lead to reproductive disorders in male animals, thus decreasing reproductive capacity [4,5]. The critical site of spermatogenesis is the testis, which, as an essential reproductive organ in male animals, contains a variety of spermatogenic epithelial cells that play a vital role in the secretion of androgens and the formation of spermatozoa [6,7,8]. Therefore, studying histomorphologic changes in the testis and the developmental pattern of spermatogenic cells is essential for controlling the reproductive process and regulating the fertility of animals. To date, study of testicular and spermatogenic epithelial cell development to control the developmental and reproductive stages of animals has been widely undertaken in mice, cats, pigs, sheep, and other animals [9,10,11,12]. In our research, we applied a similar approach to the genus Equus.

Transcriptome refers to all products transcribed by a cell at a particular stage, including coding and non-coding RNAs [13,14,15]. When the same cell is located in different life processes and growth environments, its gene expression status varies. This explains the specificity of transcriptome studies, i.e., of the existence of multidimensional differences in time and space, which, in turn, has made it possible to identify large and complex regulatory networks among genes [16]. In-depth exploration of transcriptomic information is crucial for interpreting interactions within gene regulatory networks. Single-cell RNA sequencing (scRNA-seq) refers to the high-throughput sequencing of mRNA at a single-cell level, based on the characteristics of single cells and high throughput [17,18]. It solves the problems of cell heterogeneity and small cell volume that prevent conventional high-throughput sequencing in the study of cellular molecular mechanisms [19,20,21]. With the development of single-cell transcriptome sequencing technology, more and more researchers have begun introducing this technology into animal reproduction, which has led to a gradual deepening of the study of germ cells. Single-cell transcriptome sequencing technology can be used to identify types of germ cells, categorize populations, and screen various vital genes and pathways [22].

The Mongolian horse as an excellent breed; it is one of the most famous local breeds in China and even in the whole world. It is a ride-and-drag type breed, with strong adaptability and resistance to harsh climates and rough feeding conditions [23,24]. In view of its excellent economic and cultural value, Mongolian horse germplasm conservation has become a research hotspot. Therefore, we set out to study the tissue, cellular, and molecular differences in Mongolian horse testes before and after sexual maturity by means of histomorphology and single-cell transcriptome sequencing, with a view to screening marker genes for the identification of Mongolian horses before and after sexual maturity.

## 2. Materials and Methods

### 2.1. Paraffin Sectioning of Testicular Tissue

Testicular tissue samples were obtained from Mongolian horses sourced from the Equus Research Center at Inner Mongolia Agricultural University, Hohhot, China. For this study, we selected Mongolian horses of two distinct age groups: 1-year-old (12 months) and 10-year-old. The selection of horses was based on their ages, which were determined primarily through management records and were further verified by examining their teeth, a common and reliable method for estimating a horse’s age [25,26]. Testicular tissue collection was conducted after euthanasia of the animals. All sampling procedures were approved by the Laboratory Animal Welfare and Ethics Committee of Inner Mongolia Agricultural University (Approval Code: NND2022046) and complied with the required standards. The euthanasia process ensured minimal suffering to the animals; the tissue samples were collected immediately afterward, adhering strictly to ethical guidelines and animal welfare considerations.

After collecting the samples, the testicular tissue was fixed at 4 °C for 24 h and then gently rinsed with running water to ensure complete removal of the fixative (4% paraformaldehyde) (Solarbio Science & Technology Co., Ltd., Beijing, China) before dehydration. Gradient dehydration was performed using ethanol, specifically, 0.5 h in 75% ethanol I (Solarbio Science & Technology Co., Ltd., Beijing, China), 1.5 h in 75% ethanol II, 1 h in 80% ethanol, 1 h in 85% ethanol, 1 h in 95% ethanol, and 1 h in absolute ethanol, totaling 6 h. Subsequently, the dehydrated tissues were placed in a mixed solution of xylene (Kemao Chemical Reagent Co., Ltd., Tianjin, China) and absolute ethanol at equal volumes for 20 min, followed by 10 min in xylene for clarification. After clarification, an appropriate amount of paraffin (Solarbio Science & Technology Co., Ltd., Beijing, China) was placed in a beaker and repeatedly melted and solidified in a 68 °C oven to ensure the complete removal of bubbles from the wax. The tissues were then immersed in a mixed solution (xylene: paraffin = 1:1) for 2 h. Following this, the container was changed, and the tissues were immersed in paraffin I for 1 h and paraffin II for 2 h (in a 68 °C oven) before embedding. The embedded tissues were sectioned and mounted on slides using a microtome. The slides (Leadtech Scientific Instruments Co., Ltd., Shanghai, China) were then baked in a 37 °C incubator for 5 h and stored for future use.

### 2.2. Hematoxylin and Eosin (H.E.) Staining

The prepared paraffin sections were sequentially placed in Xylene I for 5 min and Xylene II for 5 min for dewaxing. Following dewaxing, gradient hydration was performed using ethanol, specifically, 5 min in absolute ethanol and 2 min each in 95%, 85%, and 75% ethanol and distilled water. Subsequently, the sections were immersed in hematoxylin stain (Solarbio Science & Technology Co., Ltd., Beijing, China) for 6 min for staining. After staining, differentiation, and bluing were performed using a hydrochloric acid–alcohol differentiation solution and bluing solution (Solarbio Science & Technology Co., Ltd., Beijing, China), respectively. Following rinsing with tap water, the sections were then immersed in eosin stain (Solarbio Science & Technology Co., Ltd., Beijing, China) for 5 min for staining. Post-staining, the sections were soaked in tap water for 5 min and dehydrated by soaking twice in 95% ethanol, absolute ethanol, and xylene for 1 min each. Finally, the sections were mounted with neutral balsam and observed under a light microscope (Olympus Sales & Service Co., Ltd., Tokyo, Japan) for H.E. staining evaluation.

### 2.3. Single-Cell Transcriptome Sequencing

Cell quality inspection: The testicular tissue samples from Mongolian horses underwent tissue dissociation to prepare a single-cell suspension. Following a cell quality assessment, the cell concentration of the suspension was adjusted to reach a range of 700–1200 cells/µL and 10× tagged cDNA fragments. A mixture containing gel beads with barcode information was combined with cells and enzymes. This mixture was then encapsulated using oil-surfactant droplets within a microfluidic system to form Gel Bead-in-Emulsions (GEMs). The GEMs flowed into a reservoir and were collected. The gel beads dissolved, releasing the barcode sequences, which were used for reverse transcription of the cDNA fragments, thereby labeling the samples. The gel beads were disrupted and the oil droplets broken, followed by PCR amplification using the cDNA as a template. The products from all GEMs were pooled to construct a standard sequencing library as follows: Initially, the cDNA was fragmented into sections of approximately 200–300 bp. Traditional second-generation sequencing library preparation procedures, such as the addition of sequencing adapters and primers, were followed. Finally, PCR amplification was performed to obtain a DNA library. The prepared library underwent high-throughput sequencing using the paired-end sequencing mode of the Illumina sequencing platform [27,28,29] (Illumina, Inc., San Diego, CA, USA).

### 2.4. Bioinformatics Analysis

The Cell Ranger pipeline (7.1.0, https://www.10xgenomics.com, accessed on 25 October 2023) was utilized to process the raw data, which involved data filtering, genome alignment, transcriptome quantification, and cell identification. This comprehensive approach yielded a gene expression matrix for each individual cell. Subsequently, Seurat (4.1.1, https://satijalab.org/seurat/, accessed on 25 October 2023) was employed for further cell filtering, normalization of cellular expression quantities, subclassification of cellular subpopulations, analysis of differentially expressed genes within each subpopulation, and the screening of marker genes. Additionally, R (4.1.2, https://cloud.r-project.org, accessed on 26 October 2023) was used to identify the cell types within the clusters obtained from sequencing data and to perform single-cell data mining [30,31,32].

### 2.5. RNA Extraction and Reverse Transcription

mRNA was extracted using the TRlzol method. The mRNA extracted from tissue samples was then strictly reverse transcribed using the PrimeScript™ RT Master Mix (Perfect Real Time) kit (TaKaRa Bio Inc., Dalian, China) [33,34] (Table S1).

### 2.6. Real-Time Quantitative PCR

Using Primer (5.0) software, primers were designed based on reference sequences from NCBI (https://www.ncbi.nlm.nih.gov, accessed on 16 November 2023) and subsequently synthesized by Sangon Biotech Co., Ltd. (Shanghai, China). Glyceraldehyde-3-phosphate dehydrogenase (GAPDH) was selected as the internal reference gene for real-time quantitative PCR, with three technical replicates. Real-time quantitative PCR was achieved using a fluorescent quantitative PCR detection system (BIO-RAD, Hercules, CA, USA). The relative gene expression levels were calculated using the 2^−∆∆Ct^ method [35,36] (Tables S2 and S3).

### 2.7. Immunohistochemical Staining

After deparaffinizing the paraffin sections in water, the tissue sections were placed in a repair box filled with citrate antigen repair buffer (pH = 6.0) (Beyotime Biotechnology Co., Ltd., Shanghai, China) and washed three times with PBS (Phosphate Buffered Saline) (Solarbio Science & Technology Co., Ltd., Beijing, China). The washed sections were then incubated in a 3% hydrogen peroxide solution (Beyotime Biotechnology Co., Ltd., Shanghai, China) at room temperature in the dark for 25 min for antigen retrieval. They were then washed three times with PBS and treated to block endogenous peroxidase. Following blockage, a histological pen (Beyotime Biotechnology Co., Ltd., Shanghai, China) was used to draw a circle around the tissue, and 3% BSA (3% Bovine Serum Albumin) (Beyotime Biotechnology Co., Ltd., Shanghai, China) was applied to uniformly cover the tissue for blocking at room temperature for 30 min. After serum blocking, the primary antibodies, specifically DAZL Polyclonal Antibody (Thermo Fisher Scientific Inc., Waltham, MA, USA), diluted 1:200 in PBS, and PGP9.5 Monoclonal Antibody (BH7) (Thermo Fisher Scientific Inc., Waltham, MA, USA), diluted 1:500 in PBS, were applied dropwise onto the sections. The sections were then incubated overnight at 4 °C in a humidified chamber. The following day, the appropriate secondary antibody (HRP-labeled) was added dropwise to the circled area and incubated at room temperature for 50 min. Finally, DAB (Diaminobenzidine) (Solarbio Science & Technology Co., Ltd., Beijing, China) was used for color development, followed by counterstaining of the nuclei, dehydration, and mounting. A bright-field microscope (Olympus Sales & Service Co., Ltd., Tokyo, Japan) was used for section observation [37,38].

### 2.8. Immunofluorescence

After dewaxing the paraffin sections in water, antigen retrieval was performed by heating the sections in an EDTA antigen retrieval buffer, i.e., ethylenediaminetetraacetic acid (Beyotime Biotechnology Co., Ltd., Shanghai, China), followed by washing with PBS. To eliminate autofluorescence, a circle was drawn around the tissue and a quenching agent for autofluorescence was added, which was then rinsed with running water. Serum blocking was achieved by incubating the sections with BSA. Subsequently, the primary antibodies, specifically DAZL Polyclonal Antibody (Thermo Fisher Scientific Inc., Waltham, MA, USA), diluted 1:200 in PBS, and PGP9.5 Monoclonal Antibody (BH7) (Thermo Fisher Scientific Inc., Waltham, MA, USA), diluted 1:500 in PBS, were applied dropwise onto the sections, which were then incubated overnight in a humidified chamber. After overnight incubation, the sections were washed with PBS, and the secondary antibody was applied within the circled area. This was followed by counterstaining the nuclei with DAPI (4′, 6-diamidino-2-phenylindole) (Beyotime Biotechnology Co., Ltd., Shanghai, China), washing the sections with PBS, and applying DAPI stain within the circled area. Finally, the sections were mounted using an antifade mounting medium and stored in a slide box for observation under a fluorescence microscope [39] (Olympus Sales & Service Co., Ltd., Tokyo, Japan).

### 2.9. Western Blot

Proteins were extracted from the samples using an extraction buffer, followed by quantification of the total protein content in the extracts using a standardized method. The extracts were then subjected to electrophoresis to separate the proteins, which were subsequently transferred onto a nitrocellulose membrane via blotting. After blocking non-specific binding sites on the membrane, the primary antibodies were applied. Specifically, DAZL Polyclonal Antibody (Thermo Fisher Scientific Inc., Waltham, MA, USA) was diluted at a ratio of 1:2000 in PBS, while PGP9.5 Monoclonal Antibody (BH7), (Thermo Fisher Scientific Inc., Waltham, MA, USA), was diluted 1:5000 in PBS. Both antibodies were incubated with the membrane overnight at 4 °C. Subsequently, the membrane was washed thoroughly to remove unbound antibodies, and the appropriate secondary antibody was applied. After incubation and additional washes, the proteins were detected using an enhanced chemiluminescence (ECL) detection system (Qinxiang Scientific Instrument Co., Ltd., Shanghai, China). The blots were then imaged and analyzed to determine the presence and relative abundance of the target proteins [40].

### 2.10. Statistical Analysis of Data

The experimental data were organized using Microsoft Excel 2016 software. SPSS 22.0 software was employed to conduct one-way analysis of variance (ANOVA), and the least significant difference (LSD) method was used for significance testing. The experimental data were expressed as mean ± standard error. A *p*-value greater than 0.05 indicated no significant difference, while *p* < 0.05 denoted significant difference, and *p* < 0.01 represented extremely significant difference [41,42,43].

## 3. Results

### 3.1. Histomorphometric Analysis of Testes of Sexually Immature and Sexually Mature Mongolian Horses

**Figure 1:**
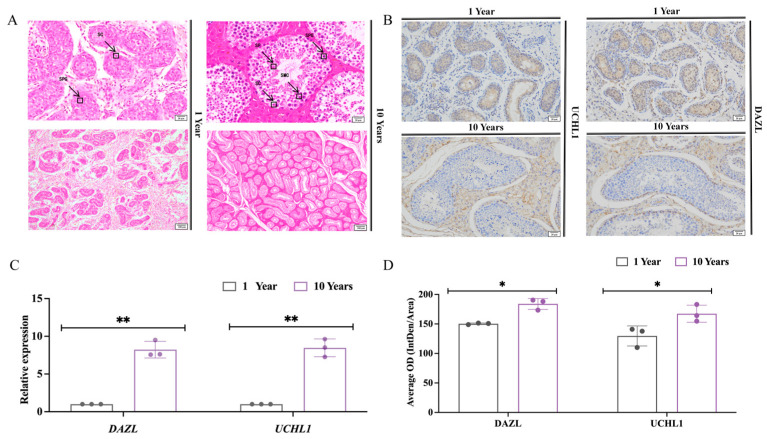
Histologic observations of the testes of unsexed and sexually mature Mongolian horses (H.E., SPG, spermatogonia; SC, Sertoli cells; SPC, spermatocyte; SP, spermatozoa). (**A**) Testicular tissue of Mongolian horses aged 1 and 10 years; scale bar 20 μm, 100 μm. (**B**) The positive expression of DAZL and UCHL1 in the testicular tissue of Mongolian horses aged 1 and 10 years; scale bar 20 μm, 100 μm. (**C**) The relative expression of *DAZL* and *UCHL1* genes in Mongolian horse testis tissue before and after sexual maturity, ** indicates *p* < 0.01. (**D**) The average OD of DAZL and UCHL1 protein in Mongolian horse testicular tissue before and after reaching sexual maturity, * indicates *p* < 0.05.

Among testicular cells, Sertoli cells are located in the basement membrane side of the wall of the seminiferous tubules, with oval or triangular nuclei that are lightly stained and have distinct nucleoli. The spermatogonia are large, round, or oval, with large nuclei and cytoplasm, and are often located in the spermatic cord basement of the testis. The spermatozoa are in the form of an elongated, curved “flagellum”, consisting mainly of a head and a tail. The head contains the genetic material and has a needle-like acrosome that helps the sperm to pass through the outer layer of the egg. The tail contains mitochondria, which provide energy support for the sperm to help it swim. A histological analysis of testicular tissues from 1-year-old Mongolian horses (Figure 1) showed the presence of many convoluted seminiferous tubules in the tissue sections of 1-year-old horse testes. Additionally, the lumen appeared but was irregular and small in diameter; the lumen did not appear to be a cavernous structure. There were Sertoli cells and a few spermatogonia, which were distributed close to the basement membrane, with small cytosol, rounded nuclei, and dark coloring. In contrast, testicular tissues from 10-year-old Mongolian horses had tubules with large diameters, and the lumen appeared to have a cavity structure. The lumen had a complete assortment of spermatogenic epithelial cells, with spermatogonia and Sertoli cells attached to the basement membrane, with a large number of spermatogonia on the inner side. A large number of spermatozoa were present in the center of the tubules (Figure 1A).

Deleted in Azoospermia-Like (DAZL) and Ubiquitin Carboxyl-Terminal Hydrolase L1 (UCHL1) are spermatogonia-related marker genes. The results of real-time fluorescence quantitative PCR (Figure 1C) showed that the relative expression of *DAZL* and *UCHL1* in the testes of 10-year-old Mongolian horses was significantly higher than that of 1-year-old horses (*p* < 0.01). Immunohistochemical results showed (Figure 1B) that DAZL and UCHL1 were significantly positively expressed in the convoluted seminiferous tubules of Mongolian horse testes at the ages of 1 and 10 years, and the average positivity rate of DAZL and UCHL1 in the testes of 10-year-olds was significantly higher than that in 1-year-olds (*p* < 0.01) (Figure 1D).

### 3.2. Classification of Single-Cell Subpopulations and Their Visualization

**Figure 2:**
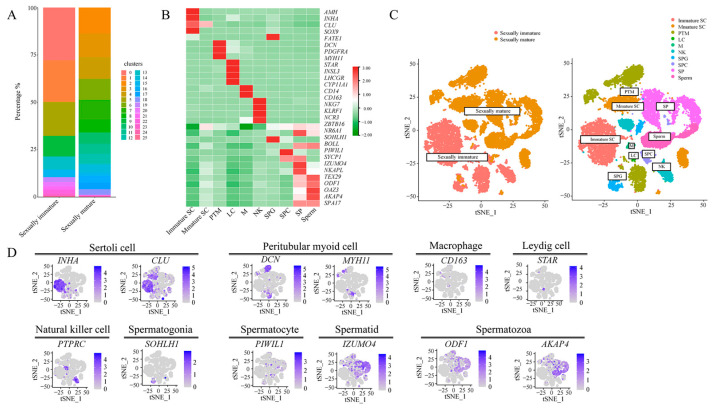
Classification of single-cell subpopulations and their visualization. (**A**) Percentage of clusters in the two samples (Y-axis); different colors represent different clusters. (**B**) Expression of selected marker genes in various cell types. (**C**) t-SNE plots of testicular cell clusters defined by scRNA-seq analysis before and after sexual maturation, colored according to cell type, with different colors representing different cell types. (**D**) The expression pattern of the selected marker genes on the t-SNE map.

The single cells were analyzed based on Seurat for dimensionality reduction clustering and subclustering into 25 clusters. Statistical tables of the number of cells in each cluster for each sample are presented in Tables S4 and S5. According to the proportion of these clusters for the histogram drawing and correlation analysis, it can be seen that the main proportion of clusters in the sexually immature (1-year-old) group was clusters 0 and 1; in the sexually mature (10-year-old) group, the main proportion was clusters 2, 3. There was therefore a difference in the distribution of the individual clusters in the two groups (Figure 2A). T-distributedstochastic neighbor embedding (t-SNE) analysis was performed on the sequencing data of all samples, and cell classification was performed using known marker genes (Figure 2B,D), which yielded a total of 10 major cell types, with the following cell types and markers: clusters 0, 1, 19, and 20 were immature Sertoli cells (Immature SC, *AMH*^+^/*INHA*^+^/*CLU*^+^); clusters 2, 10, 24, 25 were mature Sertoli cells (Mature SC, *SOX9*^+^/*FATE1*^+^); clusters 5, 7, 14, 16, 23 were peritubular myoid cells (PTM, *DCN*^+^/*PDGFRA*^+^/*MYH11*^+^); cluster 22 comprised leydig cells (LC, *STAR*^+^/*INSL3*^+^/*LHCGR*^+^/*CYP11A1*^+^); cluster 21 was macrophages (M, *CD14*^+^/*CD163*^+^); clusters 9 and 11 were natural killer cells (NK, *NKG7*^+^/*KLRF1*^+^/*NCR3*^+^/*PTPRC*^+^); clusters 13 and 18 were spermatogonia (SPG, *ZBTB16*^+^/*NR6A1*^+^/*SOHLH1*^+^); cluster 15 comprised spermatocytes (SPC, *BOLL*^+^/*PIWIL1*^+^/*SYCP1*^+^); clusters 4, 6, and 8 were spermatids (SP, *IZUMO4*^+^/*NKAPL*^+^/*TEX29*^+^); and clusters 3 and 12 were spermatozoa (Sperm, *ODF1*^+^/*OAZ3*^+^/*AKAP4*^+^) (Figure 2C).

### 3.3. Pseudotime Analysis and Visualization of Spermatogenic Cells

**Figure 3:**
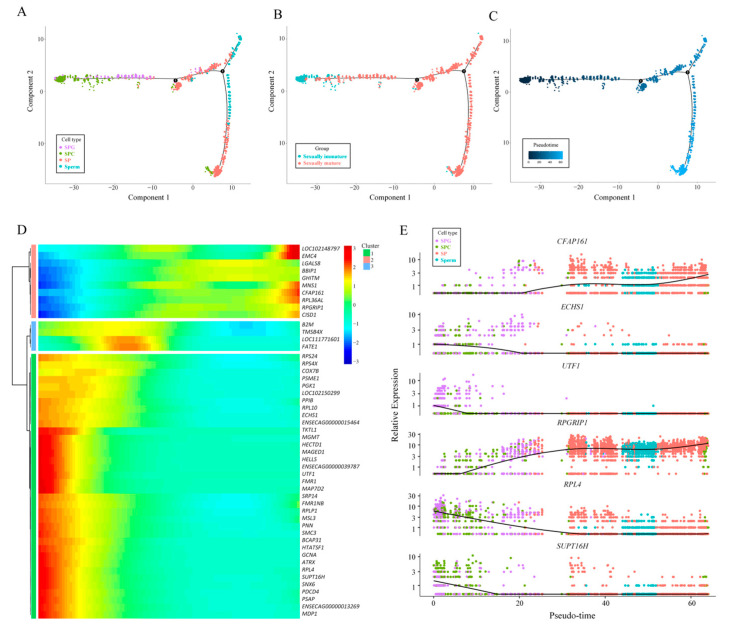
Pseudotime analysis and visualization of spermatogenic cells. (**A**) The resulting graph of proposed time series visualization based on cellular annotations. Different colors in the graph indicate different cell types. (**B**) Graph visualizing time series based on sample type annotations. Different colors in the graph indicate different sample types. (**C**) Diagram of the time trajectory of differentiation at which the cells are differentiated. Darker colors in this plot represent the default starting point, and lighter colors indicate a greater distance from the pseudo-timeline starting point. (**D**) A heatmap of the proposed chronology of genes. (**E**) Gene expression distribution maps based on cellular annotation coloring. The different colors in this figure indicate different cell subclasses, and the vertical coordinates indicate the expression level of the gene.

Based on Monocle 2′s pseudotime analysis and the visualization results, the pseudotime sequence of spermatogenic cells (SPG, SPC, SP, Sperm) started from SPG and ended with sperm, with a total of two differentiation sites. Meanwhile, different cell types occupied distinct positions along the differentiation trajectory (Figure 3A,C). As for the two samples (sexually immature, sexually mature), the pseudotime progression began with the sexually immature state and concluded with the sexually mature state (Figure 3B,C). Regarding gene expression changes over pseudotime, the varying genes were primarily grouped into three clusters with similar expression patterns. Specifically, cluster 1 (including genes like *CFAP161*, *RPGRIP1*, etc.) was predominantly expressed in the early pseudotime, cluster 2 (including genes like *B2M*, *TMSB4X*, etc.) was mainly expressed in the middle pseudotime, and cluster 3 (including genes like *ECHS1*, *UTF1*, etc.) was primarily expressed in the late pseudotime (Figure 3D). When mapping the top six most differentially expressed genes to different cell types, it was observed that cilia and flagella associated protein 161 (CFAP161) and RPGR interacting protein 1 (RPGRIP1) were predominantly expressed in SP and Sperm cells, while enoyl-CoA hydratase, short chain 1 (ECHS1), undifferentiated embryonic cell transcription factor 1 (UTF1), ribosomal protein L4 (RPL4), and SPT16 homolog, facilitates chromatin remodeling subunit (SUPT16H) were mainly expressed in SPG and SPC cells (Figure 3E).

### 3.4. Analysis of Testicular Differential Genes between Sexually Immature and Sexually Mature Mongolian Horses

**Figure 4:**
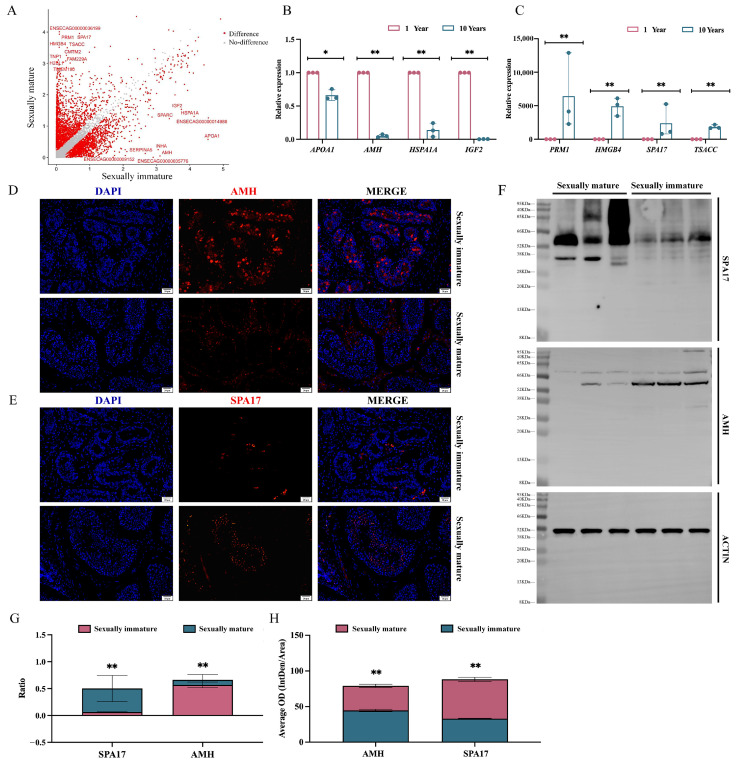
Visualization of differential gene screening. (**A**) Up-regulation and down-regulation visualization of the screened differential genes. (**B**) Relative expression of selected screened up-regulated differential genes, * indicates *p* < 0.05; ** *p* < 0.01. (**C**) Relative expression of selected screened down-regulated differential genes, ** indicates *p* < 0.01. (**D**) IF analysis of anti-AMH antibody staining on paraffin sections of testes from sexually immature and sexually mature Mongolian horses; the scale bar is 20 μm. (**E**) IF analysis of anti-SPA17 antibody staining on paraffin sections of testes from sexually immature and sexually mature Mongolian horses; the scale bar is 20 μm. (**F**) WB bands of AMH, SPA17, and ACTIN proteins. (**G**) Ration results for AMH and SPA17; horizontal coordinates are proteins, vertical coordinates are Ration. (**H**) Average oOD results for AMH and SPA17, with proteins in the horizontal coordinates and average OD values in the vertical coordinates.

Our analysis of variance was based on Seurat’s bimod algorithm with a cutoff of *p* < 0.01 and log2fc ≥ 0.26. A total of 4653 genes were identified that were significantly differentially expressed between the two groups, using sexually immature (1 year) specimens as the treatment group and sexually mature (10 years) specimens as the control group. A total of 2451 up-regulated genes and 2202 down-regulated genes were identified in the treatment group (Figure 4A). Based on the above results, the genes Apolipoprotein A1 (APOA1), Anti-Mullerian hormone (AMH), Heat Shock Protein A1A (HSPA1A), and Insulin-like Growth Factor 2 (IGF2), which were screened for significant expression in the sexually immature (1 year) group, and the genes *PRM1*, High-Mobility Group Box 4 (HMGB4), Sperm Autoantigen Protein 17 (SPA17), and *TSSK6* activating cochaperone (TSACC), which were screened for significant expression in the sexually mature (10 years) group, were subjected to the validation of real-time fluorescent quantitative PCR. The results of *AMH*, *HSPA1A*, and *IGF2*, which were expressed in the sexually immature (1 year) group, were validated by real-time fluorescent quantitative PCR. Additionally, the relative expression of *HSPA1A* and *IGF2* in the 1-year group was significantly higher than in the 10-year group (*p* < 0.01) (Figure 4B), while the relative expression of *PRM1*, *HMGB4*, *SPA17*, and *TSACC* was significantly lower than in the 10-year group (*p* < 0.01) (Figure 4C), which was in line with the trend shown in the sequencing results. Meanwhile, the immunofluorescence and WB results demonstrated that the protein level expression of AMH and SPA17 was consistent with the trend of relative expression at the gene level (Figure 4D–F and Figure S1).

The 1-year AMH ratio was significantly higher than that in the 10-year group (*p* < 0.01), while the SPA17 ratio was significantly lower than that in the 10-year group (*p* < 0.01) (Figure 4G). The 1-year AMH ratio and SPA17 ratio were significantly lower than those in the 10-year group (both *p* < 0.01) (Figure 4G). The mean fluorescence expression of 1-year AMH was significantly higher than that in the 10-year group (*p* < 0.01), while the mean fluorescence expression of SPA17 was significantly lower than that in the 10-year group (*p* < 0.01) (Figure 4H).

### 3.5. Analysis of Differential Gene Enrichment in the Testes of Sexually Immature and Sexually Mature Mongolian Horses

**Figure 5:**
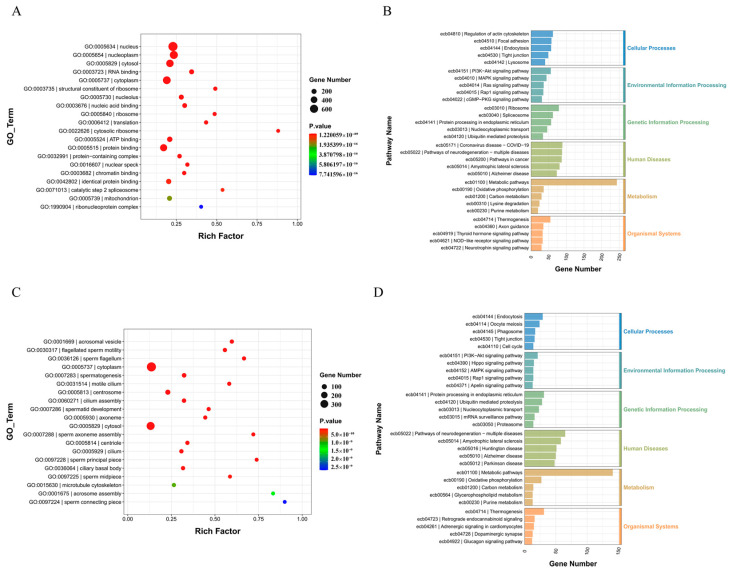
Visualization of differential gene enrichment analysis. (**A**) Up-regulation of GO terms for differential genes (*p* < 0.01). (**B**) Up-regulation of the KEGG pathway for differential gene enrichment (*p* < 0.01). (**C**) Down-regulation of GO terms for differential genes (*p* < 0.01). (**D**) Down-regulation of the KEGG pathway for differential gene enrichment (*p* < 0.01).

Gene ontology (GO) enrichment analysis was used to explore the function of differential genes in testis development and the differences between differential genes in the two groups (Figure 5A,C). A total of 612 differential GO IDs were enriched in the sexually immature (1 year) group, including “nucleus” (728 genes), “nucleoplasm” (587 genes), “cytosol” (507 genes), “RNA binding” (186 genes), “cytoplasm” (553 genes), “structural constituent of ribosome” (85 genes), “nucleolus” (191 genes), “nucleic acid binding” (163 genes), and “ribosome” (76 genes), which were predominantly enriched. A total of 233 differential GO IDs were enriched in the sexually mature (10 years) group, with “acrosomal vesicle” (37 genes), “flagellated sperm motility” (39 genes), “sperm flagellum” (32 genes), “cytoplasm” (394 genes), “spermatogenesis” (64 genes), “motile cilium” (32 genes), “centrosome” (100 genes), “cilium assembly” (50 genes), and “spermatid development” (32 genes), which were predominantly enriched. KEGG pathway enrichment analysis was performed on these differentially expressed genes (Figure 5B,D). In the sexually immature group, about 231 pathways related to “Cellular Processes”, “Environmental Information Processing”, “Genetic Information Processing”, “Human Diseases Organismal Systems”, “Metabolism”, and “Organismal Systems” were significantly enriched. In the sexually mature group, we observed about 85 significantly enriched pathways related to those mentioned above.

### 3.6. Screening for Marker Genes in Sexually Immature and Sexually Mature Mongolian Horses

**Figure 6:**
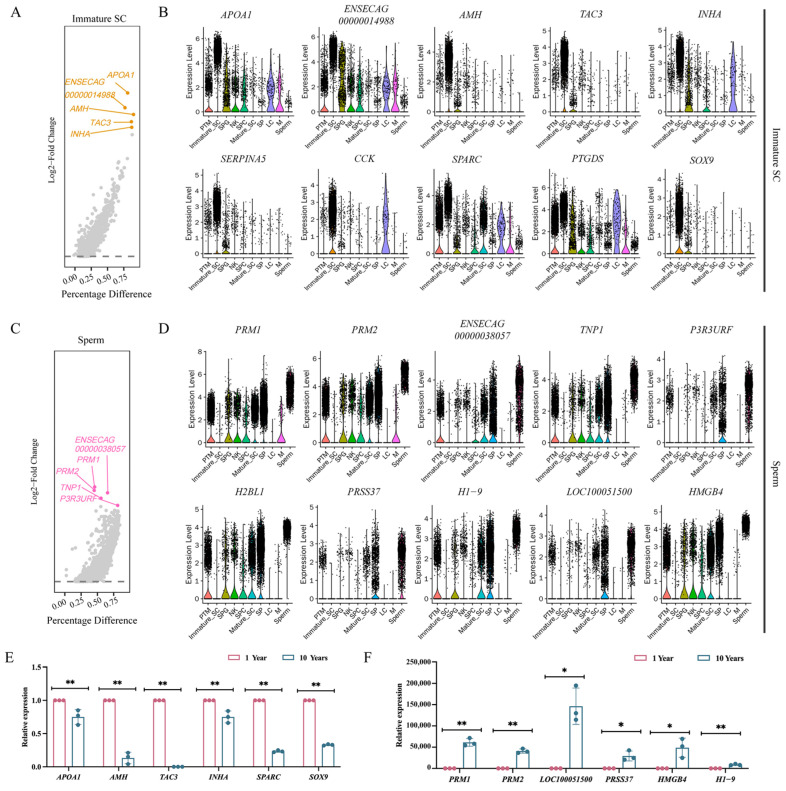
Screening of marker genes in unsexed and sexed Mongolian horses. (**A**) Map of genes specifically expressed in immature Sertoli cells. (**B**) Expression graph of the top ten immature Sertoli cell-specific expressed genes in all cell types. (**C**) Spermatocyte-specific expression gene map. (**D**) Expression graph of the top ten sperm cell-specific expressed genes in all cell types. (**E**) Real-time fluorescence quantitative PCR validation of sexually immature marker genes, ** indicates *p* < 0.01. (**F**) Real-time fluorescence quantitative PCR validation of sexual maturation marker genes, * indicates *p* < 0.05; ** *p* < 0.01.

The top ten genes screened for specific expression in sexually mature cell taxa turned out to be *APOA1*, ENSECAG00000014988, *AMH*, Tachykinin 3 (TAC3), Inhibin subunit alpha (INHA), Serpin family A member 5 (SERPINA5), Cholecystokinin (CCK), Secreted protein acidic and cysteine rich (SPARC), Prostaglandin D2 Synthase (PTGDS), and SRY-box transcription factor 9 (SOX9) (Figure 6A,B). After real-time fluorescence quantitative PCR, the relative expression of *APOA1*, *AMH*, *TAC3*, *INHA*, *SPARC*, and *SOX9* genes was significantly higher at age 1 than at age 10 (*p* < 0.01) (Figure 6E); these genes can therefore be used as markers of sexual immaturity. The top ten genes screened for specific expression in sperm taxa unique to the age of sexual maturity were Protamine 1 (PRM1), Protamine 2 (PRM2), ENSECAG00000038057, Transition Protein 1 (TNP1), PIK3R3 upstream open reading frame (P3R3URF), Histone H2B Type 1-L (H2BL1), Serine Protease 37 (PRSS37), H1.9 linker histone (H1-9), *LOC100051500*, and High mobility group box 4 (HMGB4) (Figure 6C,D). After a real-time fluorescence quantitative PCR test, the relative expression of the *PRM1*, *PRM2*, and *H1-9* genes was significant higher at 10 years than at 1 year (*p* < 0.01), while the relative expression of the *LOC100051500*, *PRSS37*, and *HMGB4* genes was significantly higher at 10 years than at 1 year (*p* < 0.05) (Figure 6F). These genes can therefore be used as markers of sexual maturity.

## 4. Discussion

### 4.1. Histomorphologic Differences in Testicular Histomorphology between Sexually Immature and Sexually Mature Mongolian Horses

The primary structure inside the testis comprises numerous twisted convolutions of seminiferous tubules that collectively perform the spermatogenic role of the testis. The histological morphology of the seminiferous tubules can visually reflect the development of the testis, the stage of development, and the reproductive capacity of the animal [44]. The results showed that when comparing the paraffin sections of the testes of Mongolian horses based on age, the cross-section of the seminiferous tubules at the age of 10 years was significantly larger than that at the age of 1 year. It was hypothesized that during the period from 1 year to 10 years of age, the testes of Mongolian horses develop rapidly under the action of the gonads, which leads to a significant increase in the diameter and area of the seminiferous tubules. Therefore, the development and growth of the testes and their internal seminiferous tubules provide the necessary conditions for the growth and development of all kinds of germ cells. This not only contributes to the formation and storage of germ cells, but also provides important support for the normal transportation of spermatozoa [45].

Various germ cells are distributed in the seminiferous tubules, among which spermatogonia play a crucial role in spermatogenesis due to their differentiation ability and stem cell potential; these cells are present at all ages [46]. The results of the present study showed that spermatogonia can be found in the convoluted seminiferous tubules of Mongolian horse testes in both the sexually immature and sexually mature stages. However, the key nodes at which the differentiation of undifferentiated spermatogonia occurs could not be accurately observed using sectioning and H.E. staining. Therefore, further in-depth studies using molecular biology are needed for observation in subsequent studies. Sexual maturity in animals describes a stage of individual development that gradually transitions to somatic maturity from the primiparous stage. Therefore, the primiparous stage is regarded as the early stage of sexually mature [47]. In the reproductive process, the primordial period, as the first critical node within it, plays a crucial role in sexual development. Subsequently, through the gradual development of the body and reproductive organs, the animal enters the sexually mature stage. In general, the age of sexual maturity among male animals exhibits species-specific variation [48]. The Mongolian horse is a primitive local breed, formed under the primitive herd grazing conditions in the alpine zone. Due to the influence of environmental and nutritional factors, its sexual maturity occurs later than in other European and American equine breeds and other species, usually entering the primordial period at about 1.5 years of age, reaching full sexual maturity after 3 years of age, and entering adulthood at the age of 5 years. The age of reproduction generally extends to about 15 years of age [49]. In this study, it was found that there were no sperm cells or spermatozoa in the convoluted seminiferous tubules of Mongolian horse testes at the age of 1 year. Sperm cells and spermatozoa appeared at the age of 10 years, so it was hypothesized that the Mongolian horses were not sexually mature at the age of 1 year and had not entered the primordial phase at that time, whereas at the age of 10 years, the Mongolian horses were already fully sexually mature. To summarize, the timing of sexual maturity depends to a certain extent on the species, breed, and other factors.

Spermatogenesis is an ongoing physiological process that is a key step in the generation of male gametes. Spermatozoa are an indispensable component in the development of a fertilized egg in mammals. In this process, various types of germ cells play a key role [50]. Griswold [51], in his study of mammalian spermatogenesis, found that the biological process of spermatogenesis has a strict cyclical pattern, i.e., the number of associated germ cells will be increased as the reproduction process continues. In this experiment, we verified the relative expression levels of the relevant marker genes (*UCHL1* and *DAZL*) in some spermatogenic epithelial cells and immunohistochemistry. It was found that *UCHL1* and *DAZL* were expressed in male germline stem cells (mGSCs). Among them, *UCHL1* is closely related to the processes of oocyte maturation, spermatogenesis, and sperm–egg binding, and is expressed in spermatogonia, while *DAZL* plays an important role in germ cell development and kinship imprinting [52,53]. The results showed that the relative expression of spermatogonia-related marker genes (*UCHL1*, *DAZL*) in testicular tissues of Mongolian horses at the age of 10 years was highly significantly higher than that at the age of 1 year, and the immunohistochemical positivity rate was significantly higher than that at the age of 1 year. This is due to the fact that in the reproductive system, spermatogenic cells are assisted by Sertoli cells and undergo a series of complex processes starting from spermatogonia, such as mitosis, meiosis, and spermatogenesis, in order to finally form mature spermatozoa. During these processes, as spermatogenesis continues and the spermatogenic cells continue to mature, they gradually change to form mature spermatozoa that are motile and capable of fertilization, and the spermatogonia continue to increase.

### 4.2. Single-Cell Mapping of Mongolian Horse Testes

Spermatogenesis in males is one of the key processes of animal reproduction, encompassing the complex transformation from spermatogonia in the seminiferous tubule epithelium to spermatids. This process is based on complex interactions between germ cells and somatic cells, so that a wide range of germ and somatic cell types tend to co-occur, i.e., complex cellular heterogeneity may be observed. This heterogeneity makes it difficult to accurately analyze and identify different cell types at different developmental stages, whereas single-cell sequencing technology can clearly and accurately identify different cell types in the testis and can also be used to further cluster different cell types according to specific research purposes. Green et al. [54] collected 35,000 cells from adult mouse tes-tes for single-cell transcriptome sequencing, identifying in the process all known types of germ cells and somatic cells, as well as two previously unidentified somatic cell types. Focused analyses delineated four taxa of spermatogonia and nine taxa of Sertoli cells, the latter of which are associated with the histologically determined developmental stages of the spermatogenic epithelial cycle. This finding provides a comprehensive knowledge base for the study of germ cell development and gametogenesis in vivo. Huang et al. [55], in order to characterize the cell types of buffalo testis, isolated cells from 3-month-old (pre-pubertal) and 24-month-old (pubertal) samples of healthy buffalo testis and identified nine spermatogonial cell types from a total of 32,847 cells obtained by 10× single-cell sequencing (immature Sertoli cells, mature Sertoli cells, undifferentiated spermatogonia, spermatogonia, early primary spermatogonia, late spermatogonia, round spermatocytes, elongated spermatocytes, and spermatids), and five somatic cells (perimyoblasts, mesenchymal stromal cells, macrophages, endothelial cells, and natural killer cells). In contrast, in our experiment, 25 cell groups were identified from approximately 17,000 cells, and four types of spermatogenic cells (spermatogonia, spermatocytes, spermatids, and spermatozoa) and six types of somatic cells (immature Sertoli cells, mature Sertoli cells, peritubular myoid cells, Leydig cells, macrophages, and natural killer cells) were characterized through the analysis of marker genes. Compared to the single-cell atlases of testes from other species, there were fewer somatic cell types, but the spermatogenic cell types were essentially similar. However, some highly differentiated spermatogenic cell types, such as spermatogonia, were not further categorized. This could be the focus of future experiments.

### 4.3. Pseudotime Analysis and Visualization of Spermatogenic Cells

Spermatogenesis is a precisely regulated process that involves the proliferation and differentiation of male germ cells, culminating in the production of spermatozoa—a phase characterized by dynamic cellular differentiation [56]. Complete spermatogenesis can be roughly divided into three stages. In the first stage, spermatogonial stem cells proliferate and differentiate into spermatocytes via mitosis. In the second stage, spermatocytes undergo two meiotic divisions, giving rise to spermatids. Finally, in the third phase, round spermatids undergo a series of morphological changes, gradually transforming from a round shape into elongated spermatids. These elongated spermatids will then undergo further metamorphosis, ultimately maturing into spermatozoa which are capable of effecting fertilization. This process represents a crucial stage in spermatogenesis, ensuring the normal function and fertilization capability of the spermatozoa [57,58]. The chronological results presented in this experiment align with the aforementioned process, specifically indicating the presence of a differentiation site before and after sexual maturity. The differentiation process prior to the first site pertains to spermatogonia, while the subsequent differentiation culminates in spermatocytes. The second critical time site emerges during the generation of a substantial amount of spermatozoa by spermatocytes, resulting in a significant production of spermatozoa.

*CFAP161* and *RPGRIP1* are two genes that are intimately linked to cilia or flagella functions. *CFAP161* encodes a protein that is potentially involved in maintaining the structural integrity and functionality of cilia and flagella, which are crucial organelles mediating sensation, locomotion, and intercellular signaling [59]. On the other hand, *RPGRIP1* encodes the RPGRIP1L protein, a component of cilia that is essential for preserving its structure and function [60]. In essence, both the *CFAP161* and *RPGRIP1* genes contribute significantly to cellular sensation, motility, and signaling by encoding proteins that are integral to the cilia or flagella. For sperm, their flagella provide movement through regular oscillations [61]. In this experiment, it was found that *CFAP161* and *RPGRIP1* are significantly expressed in SP and sperm cells. Meanwhile, the above genes are also significantly expressed in the second half of the pseudo-time-series of genes, indicating that they play an important role in sperm movement.

*ECHS1* encodes an enzyme known as membrane-bound second-type thioctic acid dehydrogenase, also referred to as enoyl-CoA hydratase. This enzyme is primarily involved in the oxidative metabolism of fatty acids within mitochondria, specifically converting unsaturated fatty acids to saturated fatty acids. Mutations in the *ECHS1* gene can potentially lead to functional abnormalities or deficiencies in this enzyme, thereby disrupting fatty acid oxidation metabolism [62]. *UTF1* encodes a transcriptional co-activator protein containing a leucine zipper domain. This protein acts as a bridge between the upstream activating factor *ATF2* and the basal transcription complex. Closely associated with chromatin, this protein is crucial for the normal differentiation of embryonic carcinoma and embryonic stem cells. Predominantly found in pluripotent cells, it can also function as a transcriptional repressor [63]. *RPL4* encodes a ribosomal protein that is a component of the 60S subunit of the ribosome, located in the cytoplasm. Ribosomal proteins play a pivotal role in protein synthesis [64]. *SUPT16H* encodes a protein that is the 140 kDa subunit of the Facilitates Chromatin Transcription (FACT) complex. This FACT complex interacts specifically with histones H2A/H2B, influencing nucleosome disassembly and transcriptional elongation, thereby contributing to chromatin remodeling and transcription [65]. In conclusion, these genes are critical in various biological processes, including cellular metabolism (*ECHS1*), transcriptional regulation (*UTF1*), protein synthesis (*RPL4*), and chromatin remodeling and transcription (*SUPT16H*). Our experiment also proved this point, i.e., these genes are essential for cell differentiation, as evidenced by their significant expression in highly differentiated cells such as spermatogonia and spermatocytes, primarily during the early stages of the pseudotime sequence.

### 4.4. Analysis of Testicular Differential Genes and Enrichment between Sexually Immature and Sexually Mature Mongolian Horses

Differential gene results showed that *APOA1*, *AMH*, *HSPA1A*, and *IGF2* were significantly expressed in sexually immature (1-year-old) Mongolian horses. *APOA1* is involved in cholesterol metabolism and cardiovascular disease [66], but its role in reproduction is unclear and needs to be further explored. *AMH* is the gene that encodes the antimitotic tubular hormone which is involved in the development and function of the reproductive system; its differential expression divides support into two distinct clusters of cells, i.e., immature Sertoli cells and mature Sertoli cells. The main feature of Sertoli cells in prepubertal male mammals is anti-miltering hormone (*AMH*), which lacks the expression of androgen receptors as well cannot traverse the blood–testis barrier [67]. *HSPA1A* is a gene encoding heat shock protein 70 which is involved in cellular stress response and cytoprotection [68]. *IGF2* is a gene encoding insulin-like growth factor 2 which is involved in embryonic development and cell proliferation; it is a key gene for cell development and proliferation [69]. At maturity (10 years), genes such as *PRM1*, *HMGB4*, *SPA17*, and *TSACC* were significantly expressed. All of these genes are closely related to the reproductive system and play key roles in testicular development, spermatogenesis, the packaging of sperm DNA, and sperm maturation [70,71,72]. In summary, for sexually immature versus sexually mature Mongolian horses, the difference in function of differential genes is huge. Similarly, in the results of our GO enrichment analysis, the main enrichment in immature specimens was in terms of cellular infrastructure, e.g., “nucleus”, “nucleoplasm”, “cytosol”, “RNA binding”, “cytoplasm”, etc., while in mature samples, the main enrichment was in terms of spermatogenesis. Maturity (10 years) is enriched in terms of spermatogenesis, such as “acrosomal vesicle”, “flagellated sperm motility”, “sperm flagellum “, “cytoplasm”, “spermatogenesis”, and so on. This indicates that before sexual maturity, the main processes associated with the animal’s reproductive potential and the related proliferation and differentiation of germ cells and somatic cells provide the necessary cellular basis for its reproductive capacity. As reproductive differentiation continues, the spermatocyte, as the most critical type of cell in the reproductive process, emerges, and its developmental differentiation and metamorphosis processes become the dominant reproductive processes after sexually mature. The late stage of spermatogenesis, which follows meiosis, represents a complex process involving significant morphological changes to the round spermatid. This final phase includes steps such as nucleosome remodeling, acrosome generation, flagellum formation, and the development of the sperm tail, among others. A study by Meistrich et al. [73] found that in mice, the process of sperm deformation can be categorized into 16 steps based on the elongation of the spermatozoa’s nuclear position and acrosome morphology. Steps 1–8 were associated with the presence of round spermatocytes that maintained a high level of activity in terms of transcriptional output, while steps 9–11 were associated with the presence of elongated spermatocytes, where the nucleus began to elongate and close the transcriptional machinery. Steps 12–14 were associated with the presence of a chromatin-enriched nucleus to form elongated spermatozoa, and steps 15–16 were associated with the presence of elongated spermatocytes that exhibited a typical hooked head and were later released into the lumen of the vas deferens [74]. The GO terms related to the final stage of spermatogenesis in our experiment included “acrosomal vesicle”, “flagellated sperm motility”, “sperm flagellum”, “spermatozoa”, “spermatozoa”, “spermatozoa”, “spermatozoa”, “sperm flagellum”, “cytoplasm”, “spermatogenesis” and other terminology, This is one of the fundamental reasons why differential genes are differentiated from sexually mature, pre-mature spermatozoa. Although the above-mentioned genes have well-established functions in spermatogenesis, the mechanism of regulation in horses has not yet been verified, and thus, further studies are needed.

### 4.5. Screening of Marker Genes in Sexually Immature and Sexually Mature Mongolian Horses

Sertoli cells play an important role in sexual differentiation, testis formation, and spermatogenesis, but do not differ in the timing and function they serve. The role of Sertoli cells in adult spermatogenesis is absent in the embryonic stage, and the transition from embryo to adulthood occurs at puberty [75]. Presumably, during early puberty, Sertoli cells undergo a fundamental change in their morphology and function, from a naive, proliferative state to a mature, non-proliferative state [76]. In the present experiment, it was also found that mature and immature Sertoli cells are two different cell populations in two different states; immature Sertoli cells were only found in the testes of Mongolian horses in the sexually immature stage. Screening for highly expressed genes in immature Sertoli cells could therefore be used as a marker for sexual immaturity in Mongolian horses. With the continuous development of testicular tissues, spermatocytes and spermatozoa appear in the testes of sexually mature Mongolian horses [77]. In this experiment, it was also found that spermatozoa were only present in the testes of sexually mature Mongolian horses. Screening of highly expressed genes in spermatozoa could therefore be used as marker for sexual maturity in Mongolian horses. After a real-time fluorescence quantitative PCR test, the relative expression of the *APOA1*, *AMH*, *TAC3*, *INHA*, *SPARC*, and *SOX9* genes was significant higher at the age of 1 year than at the age of 10 years. However, *PRM1*, *PRM2*, and *H1-9* were significantly more expressed at the age of 10 years than at the age of 1 year, while *LOC100051500*, *PRSS37*, and *HMGB4* were significantly more expressed higher at the age of 1 year. The relative expression of *PRM1*, *PRM2*, *H1-9*, *LOC100051500*, *PRSS37*, *HMGB4* at 10 years of age was significantly higher than that at 1 year of age. The results prove that the *APOA1*, *AMH*, *TAC3*, *INHA*, *SPARC*, *SOX9* genes could be used as marker genes of sexually immaturity, and *PRM1*, *PRM2*, *H1-9*, *LOC100051500*, *PRSS37*, *HMGB4* could be used as marker genes of sexually maturity in Mongolian horses.

## 5. Conclusions

In this study, we investigated the reproductive mechanisms of Mongolian horses at the tissue, cellular, and molecular levels. Utilizing histomorphological observations alongside cutting-edge single-cell transcriptome sequencing technology, a multi-faceted comparison and analysis of testicular tissues were conducted. This comprehensive methodology aimed to construct and elucidate a detailed single-cell atlas of Mongolian horse testes, delving into the intricate Pseudotime analysis process of spermatogenesis. Ultimately, this study sought to identify marker genes associated with sexual maturity in Mongolian horses, providing a solid theoretical foundation for future research endeavors in male Mongolian horse reproduction.

## Data Availability

All data are available within the article and its Supplementary Materials file or on request from the authors.

## Supplementary Materials

The following supporting information can be downloaded at: https://www.mdpi.com/article/10.3390/ani14091258/s1, Table S1: Reverse transcription reaction system; Table S2: Primer information; Table S3: Real-time fluorescence quantitative PCR reaction system; Table S4: Statistical output list of cell number in each subgroup of each sample; Table S5: Statistical output list of cell percentage of each sample in each subgroup. Figure S1: Uncropped Western blot figures.
