# Supplementary figures and images for: Single-Cell Transcriptome Sequencing Reveals Molecular Expression Differences and Marker Genes in Testes during the Sexual Maturation of Mongolian Horses
Source: Animals (Basel). 2024 Apr 23;14(9):1258. doi: 10.3390/ani14091258 (PMC11082968; doi:10.3390/ani14091258)

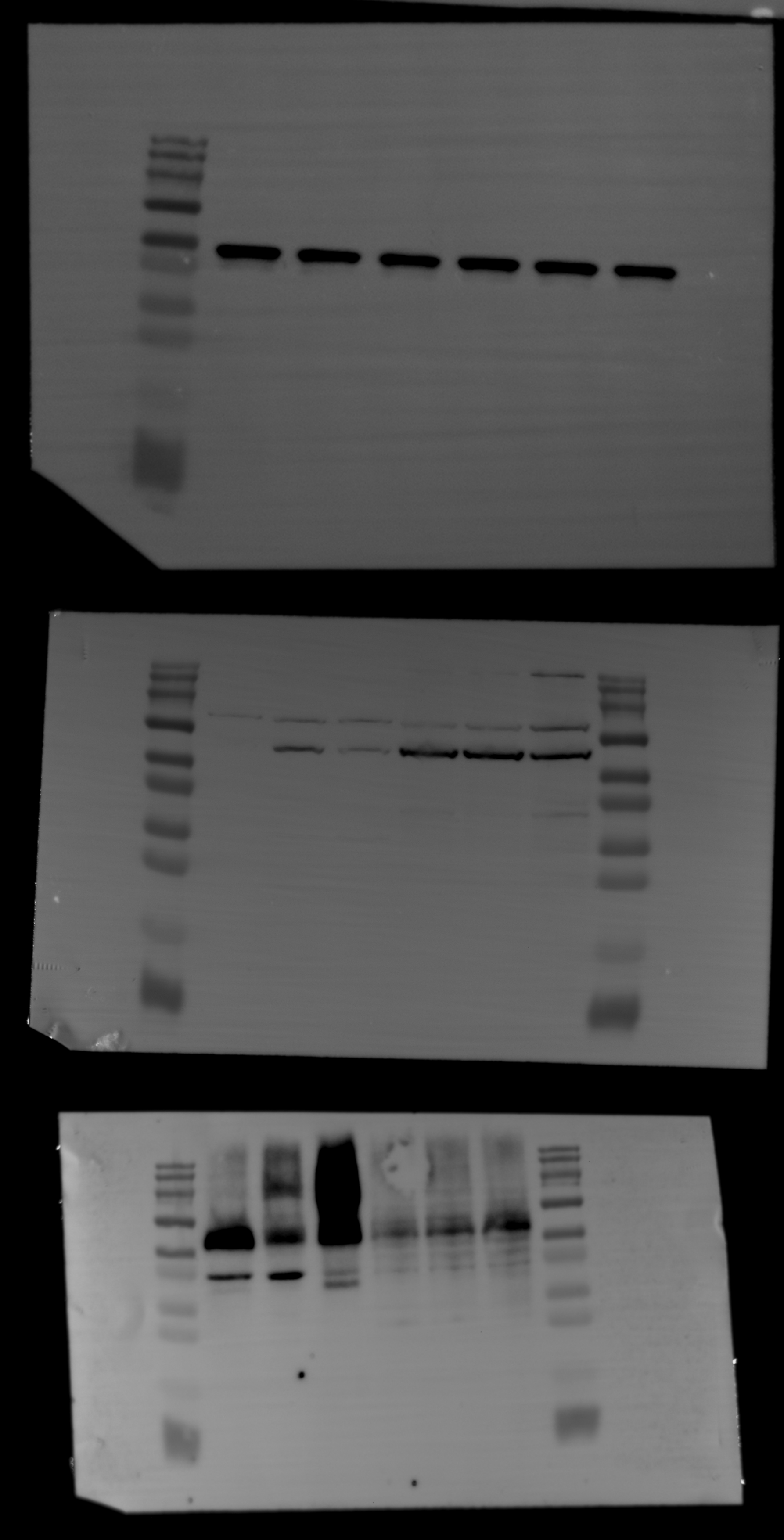

Supplement: Supplementary file 1 [file animals-14-01258-s001.zip › Figure S1 Uncropped Western blot figures.tif]
